# mGrid: A load-balanced distributed computing environment for the remote execution of the user-defined Matlab code

**DOI:** 10.1186/1471-2105-7-139

**Published:** 2006-03-15

**Authors:** Yuliya V Karpievitch, Jonas S Almeida

**Affiliations:** 1 Department of Biostatistics, Bioinformatics and Epidemiology, Medical University of South Carolina, 135 Cannon Street, Suite 303, Charleston, SC, 29425, USA; 2 Department of Biostatistics and Applied Mathematics, Dept Biostatistics and Applied Mathematics, Univ. Texas MDAnderson Cancer Center – unit 447, 1515 Holcombe Blvd, Houston TX 77030-4009, USA, USA

## Abstract

**Background:**

Matlab, a powerful and productive language that allows for rapid prototyping, modeling and simulation, is widely used in computational biology. Modeling and simulation of large biological systems often require more computational resources then are available on a single computer. Existing distributed computing environments like the Distributed Computing Toolbox, MatlabMPI, Matlab*G and others allow for the remote (and possibly parallel) execution of Matlab commands with varying support for features like an easy-to-use application programming interface, load-balanced utilization of resources, extensibility over the wide area network, and minimal system administration skill requirements. However, all of these environments require some level of access to participating machines to manually distribute the user-defined libraries that the remote call may invoke.

**Results:**

mGrid augments the usual process distribution seen in other similar distributed systems by adding facilities for user code distribution. mGrid's client-side interface is an easy-to-use native Matlab toolbox that transparently executes user-defined code on remote machines (i.e. the user is unaware that the code is executing somewhere else). Run-time variables are automatically packed and distributed with the user-defined code and automated load-balancing of remote resources enables smooth concurrent execution. mGrid is an open source environment. Apart from the programming language itself, all other components are also open source, freely available tools: light-weight PHP scripts and the Apache web server.

**Conclusion:**

Transparent, load-balanced distribution of user-defined Matlab toolboxes and rapid prototyping of many simple parallel applications can now be done with a single easy-to-use Matlab command. Because mGrid utilizes only Matlab, light-weight PHP scripts and the Apache web server, installation and configuration are very simple. Moreover, the web-based infrastructure of mGrid allows for it to be easily extensible over the Internet.

## Background

The term 'grid' was coined in the mid-1990s and designates distributed computing infrastructures for science and engineering [1]. Today, Grids seek to provide seamless, scalable, high-performance and secure mechanisms for locating and negotiating access to remote and possibly heterogeneous resources [2,3]. The specific goal underlying the Grid concept is coordinated resource sharing and problem solving in dynamic, multi-institutional virtual organizations [4].

Computational biology applications with large memory and CPU requirements have been a significant driving force behind grid development [2,5-9]. However, the discovery mode of many computational biology initiatives which favours the autonomous use of rapid prototyping environments by small groups or even individual researchers, which does not bode well with the predominant, heavier, distributed computational infrastructures available. In this regard it is useful to recall the context of their development and in particular the eroding distinction between data and computational grids.

Some grid middleware has evolved to bind data resources scattered across a network into a unified environment to make it appear to the user as if all the data is stored locally on that user's desktop (data grids). Computational grids, on the other hand, provide the ability to perform computations remotely, possibly in parallel, without the user having to know anything about networking and communication between the computers (distribution transparency). Majority of grids today act as both data and computational grids [6-9].

Particularly illustrative examples of working grid systems for computational biology are Folding@home, North Carolina BioGrid, caBig, and myGrid. The goal of Folding@home is understanding protein folding, protein aggregation, and related diseases by utilizing idle CPU cycles (i. e. computational grid) over a collection of computers [5]. NC BioGrid combines computing and data resources into a unified environment for genomic research (data and computational grid) [8]. caBig is the cancer Biomedical Informatics Grid, it is made up of network of individuals and institutions to enable the sharing of data and tools for cancer research [9,10]. Another example of the grid that is being used in bioinformatics is myGrid [11,12]. myGrid is an infrastructure specifically designed to support biological applications. It provides access to analysis tools (computations) or database services thus making it a diverse service grid. Although the above implementations are excellent examples of the working bioinformatics grids, their scope is far beyond the scope of the solution proposed in this report, and most importantly none of these grids allow for distribution of user-defined libraries on remote machines at runtime, which is the main goal of mGrid.

Matlab is a particularly popular computing environment for rapid prototyping [13]. The potential advantages of building a distributed computing capability in Matlab were reflected by the development of over twenty infrastructures to distribute and/or parallelize Matlab [14-19]. Some examples are: MultiMatlab, MatlabMPI, MATLAB*G, Distributed Computing Toolbox. Most of the existing tools require an underlying parallel library or language, such as Message Passing Interface (MPI) [20], Parallel Virtual Machine PVM [21], or a communication technology like Jini [22] or ALiCE [2]. The installation and configuration of such environments often require a significant level of system administration knowledge and makes systems less secure. Also, message-passing and shared-memory tools are designed to work best on a local area network (LAN), for example on a cluster of machines. These tools are not easily extensible to span across large heterogeneous wide area networks (WANs) such as the Internet [23,24]. Moreover, none of these environments allow for distribution of user-defined Matlab libraries.

It is noteworthy that our method relying on code distribution is not proposed in strict alternative to message passing. Quite the opposite, there is no fundamental reason why they cannot be mixed.

Some environments only allow for coarse-grained parallelization, i.e. at the functional level or only parallelization of matrix operations and for-loops [15,18]. For example, the Distributed Computing Toolbox [15] is a Matlab native toolbox that enables users to execute independent parallel algorithms on a set of machines simultaneously. This library, which works together with the Distributed Computing Engine, requires Jini technology to facilitate communication between computers that comprise a distributed computing environment. Jini is a Java based software and thus requires installation of the Java specific runtime environment and Jini servers on all machines. Once again, the Distributed Computing Toolbox does not allow for the dynamic distribution of the user code.

The differentiating characteristic of mGrid is indeed user code distribution. While this report focuses on a proof-of-concept of Matlab implementation of code distribution, the concept itself is general and can be embedded in other programming environments. mGrid allows unique user code to be dynamically migrated to heterogeneous computers, i.e. it allows for user-defined Matlab commands and libraries of functions needed to execute those commands, and run-time variables, to be passed to the remote machines automatically. This involves run-time allocation of resources, such as, available CPU cycles, memory, and user code and data distribution.

A reliable file transfer capability is provided by GridFTP which could be used for transferring the necessary user code in mGrid. Using GridFTP for our purposes would require the installation of both the GridFTP client on the client machines and the GridFTP server on the worker systems. Furthermore, the worker machines must also have the Globus Toolkit installed. The authors believe that these installation requirements are overly complicated and have decided to implement a simpler solution using PHP [25] and hyper text transfer protocol (HTTP).

A second feature of mGrid is support for parallel remote Matlab command execution. Thus, mGrid includes the ability to distribute computations in parallel, although in a basic sense, i.e. what is commonly called "embarrassingly parallel" computations [23]. In the code distribution by mGrid this is achieved by coarse-grained parallelization, at the function level.

The specific goal of this research was to advance the automatic distribution of algorithms written in Matlab by developing a library for distributed computing on blade servers at the Bioinformatics Core of the NHLBI Proteomics Center at MUSC. The original motivation was to speed the reverse engineering of biological networks using S-Systems [26,27] by allowing numerical decoupling of the ensuing system of coupled differential equations [28,29]. The bioinformatics justification and likely usefulness of the code distribution scheme proposed here is a reflection of the particular need to systemically integrate a variety of data and models [30] which themselves may change in structure and even in purpose over time. Furthermore, tackling the systems biology challenge is increasingly not just a matter of coping with large amounts and large variety of data and applications but also of testing alternative theoretical foundations. Consequently, the challenge that mGrid seeks to address is the creation of a user-friendly programming environment tailored to rapid prototyping such that algorithm and theory development could share a common programming environment.

## Implementation

### The 'm' in mGrid

The letter 'm' in mGrid refers to Matlab, a programming environment by Mathworks Inc. [31], the implementation language of the mGrid software and also the language of the code being distributed. The choice of Matlab to implement code distribution is a reflection of its popularity for rapid prototyping of scientific and engineering applications. The combination of user-friendliness and support for high end applications makes it a natural choice for the implementation of grid computing through code distribution. Nevertheless, it should also be noted that Matlab is a commercial product witch raises a cost barrier to access and scaling up the proposed implementation. Nevertheless, there are free, open-source, Matlab-like tools available (Octave, FreeMat, SciLab, Tela, Euler [32-37]) onto which the implementation described here can be easily ported. Other then the language itself, this is an open source, freely distributed tool (see Availability).

Matlab was chosen for being the most complete of these environments and for the availability of implementations for all the major operating systems. The same is true for the other components of mGrid – Apache web server package and its PHP module. Therefore, the grid computing implementation described here is truly platform independent. Finally, this is also the environment of choice for training in scientific computing and computational statistics in academia.

### Connectivity

At the heart of every Grid is a network that links together distributed resources. It is important to note that mGrid uses the standard HTTP protocol which runs on top of TCP/IP, which facilitates interoperability, extensibility and portability of the software. TCP/IP also provides reliable delivery of the data, as it automatically detects errors and requests retransmission until all data is received. mGrid uses the Apache web server [38] to establish communication with contributing computers, and PHP [25]to structure the data transferred between participating computers.

### mGrid architecture

**Figure 1:**
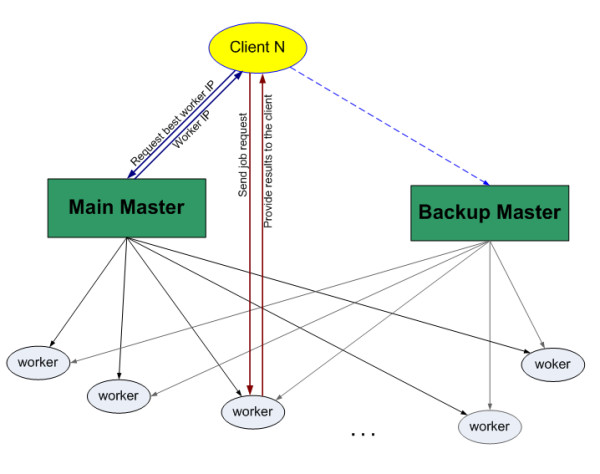
**mGrid architecture and client communication. **Architecture of full mGrid implementation: load management and multiple, parallel, remote calls are mediated by dedicated mGrid master server machines upon receipt of a global evaluation calls (using command *geval*) by client machine. *geval *requests the IP address of the current best available worker form one of the masters, then sends the job request to the worker which IP address was returned by the master. For parallel evaluation geval will simply ask for multiple IP addresses and issue the job requests to multiple worker computers.

The full mGrid implementation includes dedicated mediating server machines to manage the client requests and select the best available workers, following a semi-hierarchical architecture (Figure 1). This architecture consists of two master servers (main and backup master servers) that can independently decide which worker to send a given request to, based on user requirements for CPU speed, available RAM, and workload.

The master servers are defined as a redundant set of two machines that maintain balanced workload distribution among workers in mGrid (Figure 1). The main and backup systems maintain identical information on the availability of the worker machines to be targeted by remote calls. Global *eval *function *geval *initially communicates with the main master server. A request will always go to the same main server. The backup master will be contacted only in the case of the main server not responding to the client's request, thus preventing any downtime of the mGrid. The mGrid will be temporarily unavailable in the case of both the main and the backup master servers being down simultaneously. After geval receives the worker IP address it sends the job request to that worker.

Addition of workers to the mGrid requires a request to be sent (via the *volunteer *command in Matlab) to both master servers so that a consistent view of the worker states is maintained. Master servers have a check mechanism to prevent multiple additions of the same worker to the mGrid if *volunteer *command is executed more then once. Thus repeated executions of the volunteer command will have no effect on the masters.

Master servers maintain workload information on the workers by periodically polling the workers about the number of their available CPU cycles. This is done by *crontab *calling a PHP script update_busyinfo.php, which in turn contacts all the workers to get the workload information by calling a PHP script busyinfo.php on remote machines. The information returned includes CPU idle time, amount of available memory, and processor speed. Based on the results returned by each worker, the workload of each worker is accurately updated. If a worker becomes unavailable, then all three of the above resources are set to zero, and that particular worker will no longer be considered for incoming requests. Also, after a particular server assigns a job to a worker the workload information stored on that master server is updated by incrementing the record of the worker's workload by a constant load percentage. A load percentage of 15% empirically found to be sufficient for the purpose of statically estimating the expected workload increase on that computer and thus making it no longer the least busy worker.

The basic call to mGrid is mediated by the function *geval *(global evaluation) that selects a main master server from a list of two candidate mGrid master server machines. A message is then sent to the chosen master server requesting the IP address of the current 'best' available worker known. Upon receiving the IP address of the best available worker, *geval*, packs all the information necessary for the command to execute successfully on the remote machine:

• converts this command to a string, encoded in a format appropriate for use within a Unified Resource Locator (URL)

• encodes any workspace variables

• encodes client toolbox

*geval *then calls the *reval *procedure, which is responsible for data transfer to the remote worker(s) and collection of the results after worker(s) complete the assigned jobs.

The task of adding the master server to the mGrid needs to be performed before any other components are installed, as both client and worker installations require the address of two master servers to which they relay their requests and accept reassignments to worker machines. On the contrary, as mentioned before, dynamic accommodation of new participants is provided by the master servers so the constellation of worker machines available to the master servers for assignment of incoming jobs, can be changed at anytime without interruption of ongoing jobs or submission of new jobs. Two additional functions distinguish master servers from the workers: add_worker.php and get_worker.php. The first function, add_worker.php, allows a new worker machine to be added to the grid. This function verifies that the worker is accessible via HTTP (on top of TCP/IP) protocol and that it is ready to accept the remote jobs. Function get_worker.php is used by mGrid to provide client with the IP address(es) of the current best available worker(s).

At this time mGrid does not incorporate a client-control system. Control is managed by the network configuration, currently mGrid is located behind a university firewall preventing outside access. Anyone within the university can be an mGrid client. Virtual Private Network (VPN) can be set up to enhance security by utilizing data tunnelling and encryption to work over the Internet.

## Results

### User-defined code distribution

User code distribution is the defining feature of mGrid, allowing a user to provide and operate a new function or a library of functions that is not kept on the grid itself. mGrid supports client functionality to pack the user's unique code and variables, transfer them over a network to a remote worker machine where they are unpacked and evaluated. This makes mGrid a desirable tool for scientists that write their own functions and want them to be executed on one or more remote computers without having to install those functions on all of the machines participating in the mGrid. This is a convenient and more secure feature than process distribution, because it eliminates the need to require that users have permissions to install or write files on the distributed computers. It also eliminates having to propagate every small user code changes to all of the machines.

A client can issue a command to a specific worker machine, possibly passing local work space variables and functions or entire Matlab toolboxes necessary for computation. In this basic remote evaluation mode of operation (not the optimal setting as detailed later), the worker machine autonomously handles the request, makes results available to the client and then removes the client code from its hard drive, following a standard client-server model [24]. mGrid automatically handles the entire process such that user specification of parallel execution does not depend on the code, unlike other parallel environments like MPI or PVM [20,21,23] that require users to learn a parallel programming language in order to write code that can be run in parallel.

mGrid provides a fully parallel configuration where the user simply has to list the multiple commands to be executed in an array. Both the selection of worker machines and code distribution are automatically mediated by mGrid. Of course, for parallel execution, the multiple Matlab commands given by the user have to be independent of each other [23].

### Client side of mGrid

In order to access an existing mGrid infrastructure, a potential client needs only to have a small Matlab toolbox and learn how to use three new commands. As any other Matlab function, these functions can be inserted anywhere into other scripts and functions in order to have the corresponding input argument command expression evaluated by mGrid.

#### Using mGrid

From the user's perspective, the toolbox is simple to install (just download) and use. It runs on Windows, Macintosh and Linux systems. Clients wanting to utilize mGrid on a computer with a current copy of Matlab, must perform the following steps:

1. Download 'mGrid client toolbox.

2. Start using mGrid.

The toolbox comes with a text file, *masterservers*, listing the IP addresses of the two mGrid servers (the main machine and its backup, see Implementation section) available to that user. This text file can be generated by the system manager and made available to the users as a separate download or the user can edit its contents to include the correct IP addresses for the target mGrid server installation.

#### Client toolbox functions

**Figure 2:**
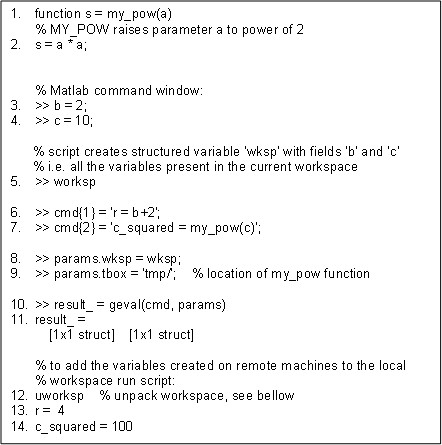
**Code example. **A client constructs proper parameters to the *geval *procedure and executes it. Upon receiving the results client unpacks resulting variables into the local workspace.

There are only three functions that need to be learned to use mGrid, though the client toolbox consists of approximately twenty:

**geval **– global *eval *procedure, evaluates command(s) on remote machine(s) participating in mGrid (line 10, Figure 2).

**worksp **– Matlab script, packs workspace variables into one structured variable *wksp *(line 5, Figure 2).

**uworksp **– Matlab script, places fields of the structured variable *result_ *into workspace (line 12, Figure 2).

Figure 2 shows an example of using mGrid. It illustrates the ease of using function *geval *to specify a list of commands to be executed in parallel (in this case 2 commands). If the first parameter is a cell array of commands, *geval *will execute them in parallel. Second parameter is a structures variable which allows the user to pass user-defined code, workspace variables and memory requirements to the grid. Memory requirement allows user to specify if larger amounts of random access memory (RAM) is needed, otherwise the CPU speed and its idle cycles will be used for deciding on the best available worker for a particular request. Second parameter can be omitted or only parts of it may be specified depending on user needs.

Because of the simple design of mGrid, the user is required to read very little documentation in order to begin writing parallel Matlab computations. Therefore, the user manual is minimal.

#### Return variables

After the completion of a *geval *procedure one structured variable, *result_*, is returned to the client with the new variables or new values of original variables. The return value should be assigned to the variable *result_ *if the use of the script *uworksp *to automatically unpack the values to the client's local workspace, is desired.

### Server side of mGrid

The configuration of an mGrid facility requires the configuration of master server and worker machines. Both are fairly simple to follow. The worker configuration in particular is designed to be accessible to the average Matlab user wanting to contribute a machine to a local mGrid infrastructure. This requires minimal system administration expertise. At this time, automated server side installers are only provided for Linux machines. Client side is platform independent. A worker can be a client of mGrid at the same time.

#### Adding a worker to the mGrid

To ensure that mGrid can dynamically accommodate new participants across different platforms, the worker side toolbox includes a function to mediate volunteering their CPU-cycles: any computer with a copy of Matlab and a web server can be made a worker without interruption or reconfiguration of ongoing processes. The automatic configuration of a new worker consists of three easy steps:

1. Download the 'mGrid Worker Toolbox'.

2. Execute the *workerconfig *script. Upon completion the Matlab function *todoprocessor *will be running as a background process.

3. Execute the *volunteer *command from Matlab, upon completion check for either an error or success message displayed. Once a success message is displayed, the worker system is ready to accept remote jobs.

A script for automatic Apache web server configuration is available for Linux computers. The script is geared towards the default installation and configuration of Apache. If some user modifications are present, the script may not be able to perform all of its tasks. The user then will have to make sure that Apache is running and is able to access users' *public_html *directories manually.

A *todopr *is a startup script that is added to the list of jobs started upon boot up of the computer to automate the process of restarting *todoprocessor *in case of a shutdown or reboot of the machine. *todoprocessor *can also be stopped, started and restarted manually by executing the *todopr *shell script manually with proper arguments.

#### Worker toolbox functions

There are two important functions in the worker toolbox:

**volunteer **– requests that the current computer be added to the workers pool on the master servers. This function should be executed after the installation of the toolbox.

**todoprocessor **– executes commands that arrive from remote machines. See display of the help file bellow.

Function *todoprocessor *is a service started automatically at computer boot time. It locates newly arrived requests, starts the execution of the requests, updates the status of the request to 'running', and finally when request is completed it makes results available to the client.

#### Adding a master server to the mGrid

As for the worker side toolbox, the automatic installation scripts provided for the master side toolbox were written specifically for computers running the Linux operating system and the Apache web server. Equivalent versions for other combinations of other operating system / web server should be straight forward. The automatic configuration consists of three easy steps:

1. Download the 'mGrid Master Server Toolbox'.

2. Execute the *masterconfig *script. Upon completion the Matlab function *todoprocessor *will be running as a background process. At this time the master is ready to issue IP addresses for the best available workers.

3. Execute the *volunteer *command from Matlab, upon completion check for the display of either an error or success message. When a success message is displayed, the worker component of the system is ready to accept remote jobs.

The *masterconfig *script is built on top of the *workerconfig *and has additional functionality necessary for the master server configuration. Thus any master server has the full functionality of the worker and is added to the pool of worker machines by the volunteer command. If a master is not intended to participate also as a worker, then step 3 should be omitted. A master can also be removed from the pool of workers at any time by manually removing its IP address from the workers file and stopping the *todoprocessor *by executing the *todopr *script with the option stop ('./todopr stop').

**Figure 3:**
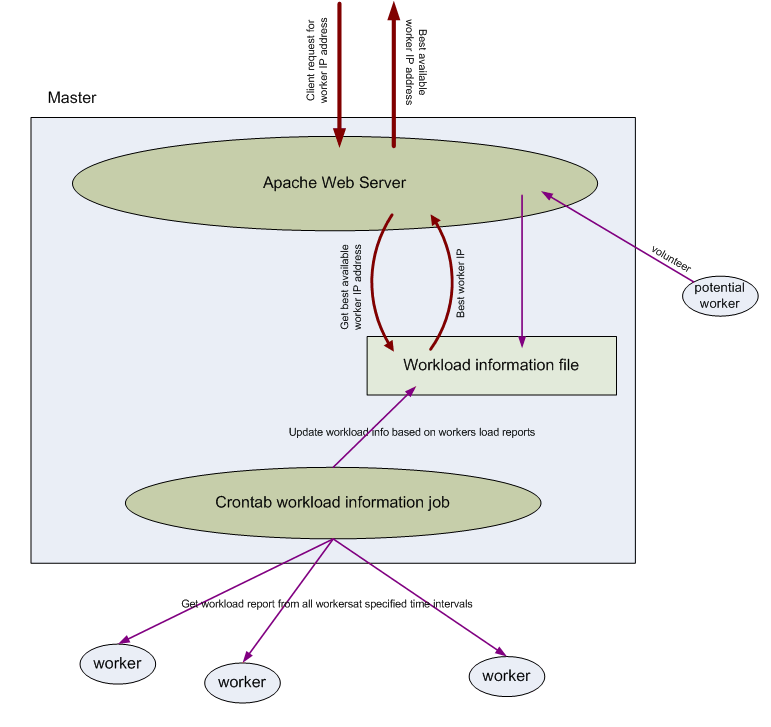
**Communication between processes on the master server. **Communication between processes on a master server (configured strictly as a master server, not also as a worker). At the top, the down pointing arrow indicates a client request for the IP of an available worker computer (geval). The Apache web server (large oval) accepts the request via a PHP script, which gets the IP address of the currently best available worker from the worker workload information file (rectangle) and returns it to the client. The worker workload file is updated periodically by a crontab job which polls the necessary information form the workers. Each new worker (outside the Master rectangle on the right) willing to participate in mGrid, executes a volunteer command, which calls a PHP script on the master node (going through Apache) to update the workers workload file. Note that if a given worker is already registered with this master, the request will simply be ignored. If a worker is new, then before adding it to the list the master will request the workload information from the potential worker and only upon successful receipt of the workload information, which verifies that the worker responds to the HTTP requests, will this volunteering computer be added to the pool of workers.

Figure 3 shows communication between processes on a master server and communication of the master server with the other machines. Apache web server accepts the requests from the clients executing *geval*, a PHP script on the master looks up the workers information in the workers' workload files and provides the IP address of the best available worker machine to the client, which sends its request directly to that worker. Best available worker is determined as a combination of the available CPU cycles, CPU speed and available memory. Computers willing to be a part of the mGrid execute volunteer command, which goes through the Apache to the PHP script which adds the volunteering computer to the list of the available workers if that computer is able to respond to the request for the workload information.

## Discussion

This paper presents an easy-to-use, configurable, distributed system that allows for parallel Matlab user code distribution. The main objective was to allow users to execute exclusive Matlab code, possibly in parallel, on a remote, perhaps more powerful, machines with minimal learning curve. To use mGrid, the user, familiar with the simple Matlab programming language, only needs to learn a single new command, the global *eval *procedure (*geval*), described in this paper. The implementation, which is based on two redundant master servers that hand down the jobs submitted to a fluid set of worker machines (Figure 1), further contributes to the user not having to be at all aware about the resources recruited to support distributed execution. Furthermore, committing new workers to mGrid is, like the code distribution itself, entirely web-based and automated through the execution of a single automatic command.

Although the initial specific motivation for the development of mGrid was to assist in experimenting with parallelizing data collocation schemes for numerical decoupling of systems of differential equations describing metabolic networks the use of code distribution was found to have a more general applicability. Consequently, mGrid was developed, as described here, with a wider range of scientific and engineering applications, within and beyond Bioinformatics. Although the implementation described is specific to the Matlab programming environment, it is simple enough to be readily portable to its open-source publicly licensed clones such as Octave and FreeMat [32,33] and to Matlab-like environments such as Euler, Tela or SciLab. [34-36]. The remaining software components of mGrid, Apache and PHP, are themselves mainstream open-source public license applications. The future development of mGrid is hereby opened to the community by releasing it under GNU General Public License (see Availability) and will likely include a version for Octave and FreeMat and a graphical user interface (GUI) with time information and various statistics on current remote executions to assist in identifying optimal load balancing schemes. Some modifications to the installation scripts are also immediately anticipated to tailor the installation to the particular design of the operating system – for example implementing *todoprocessor *as a SysV Init script (under Linux).

**Figure 4:**
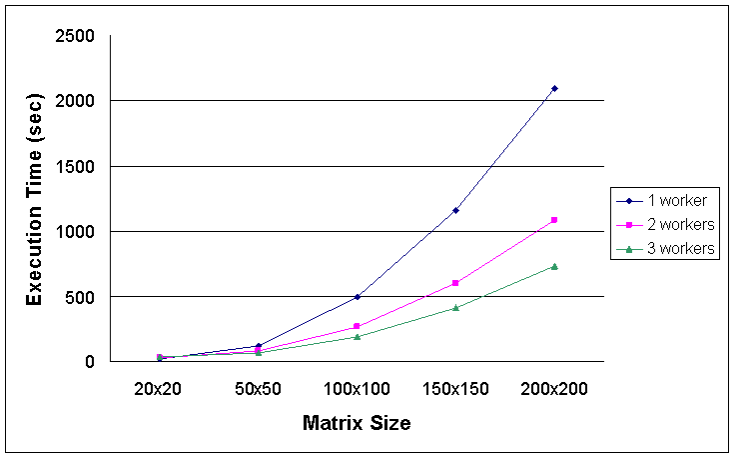
**Execution time comparison of Parzen kernels superpositioning algorithm on one, two and three computers. **The Parzen kernel is applied to a replicate series of microarrays to identify a reference conditional cumulative distribution to access statistical significance of observed differential expression. The number of possible comparisons in a replicate series with N genes and M arrays is NM-1, thus the computation can be quite time-consuming. The number of kernel evaluations is also dependent on the dimension of the resulting matrix, K, of the conditional distribution. The number of kernel evaluations would therefore be (K2.N) M-1. The speedup gained by using mGrid and separating the task into three independent subtasks for larger matrices (150 × 150 or 200 × 200) is 2.8. This is considered excellent as the maximum theoretical execution time speedup possible is 3. As the problem size increases, the data transfer time becomes relatively insignificant.

It is immediately apparent that the proposed implementation requires fast communication among participanting machines. Therefore, mGrid is not suitable for speeding up processes that would run in a few seconds on a single machine. A more suitable scenario is the one where the execution of an algorithm takes a long time to complete, thus justifying the migration of the libraries to different computers. Figure 4 displays the execution time for the identification of the density distribution using Parzen kernels for different sizes of the resulting matrix. mGrid was used to distribute this computation among two and later among three computers. The results were compared with a sample run where no distribution was used at all. Parzen kernels superimpose Gaussian "hat" distributions to determine the density distribution for a scatter plot. This is a very convenient alternative to discretizing the scale and representing the histogram of frequencies. The computation of this kernel consists of centering a Gaussian "hat" on each available point in the scatter plot. Each individual "hat" has unitary volume so the superimposed distribution can simply be divided by the number of points to get a unitary density distribution. The illustration described here applies the Parzen kernel to a replicate series of microarrays with the purpose of identifying a reference conditional cumulative distribution to access the statistical significance of observed differential expression. The rationale for this empirical solution to statistical modeling was recently illustrated by one of the authors for a proteomics dataset [39]. The superimposition of Gaussian hats is an additive procedure that can be independently computed in parallel. As can be seen from figure 4, even computations that take several minutes to complete, can benefit greatly from using mGrid. In this case, the speedup in execution time of the parallel algorithm on three computers is 2.8. This speedup is slightly less then the theoretical maximum of three, due to the overhead of user-code transfer time and remote request assembly time. Computations that can be formulated as a composition of independently computed parallel results, as exemplified by the suprapositioning of Parzen kernels illustrated above, often occurs in computational biology applications. The applications typically include multiple, independent, time consuming operations such as querying public databases, non-linear regression, and numerical simulations.

## Conclusion

Distribution of user-defined Matlab toolboxes and rapid prototyping of many coarse-grained parallel applications can now be done with a single easy-to-use Matlab command. The implementation is made available as a suite of three Matlab toolboxes, collectively described as mGrid, with client, master and worker implementations. Multiple roles can be assigned to the same machine. Because mGrid utilizes only Matlab, light-weight PHP scripts and Apache web server the installation and configuration are very simple. Moreover, the web-based infrastructure of mGrid allows for it to be easily extensible over the Internet.

The implementation of mGrid is being made freely available under a GNU General Public License [40], where contributions for further development are invited, particularly in regards to developing a version that relies entirely on public license components. A particular note regarding copyright issues is warranted for implementations relying on Mathworks Inc. Matlab environment [31]. The prototype described here relies on a set of machines served by the same concurrent license manager. For more distributed implementations attention should be paid to the details of the particular license agreement associated with the purchase. In that regard porting the implementation to Octave, FreeMat or porting the code distribution environment to support other advanced programming languages such as Python may be an alternative worth exploring. Considering the fact that all the communication is done via PHP scripts, modifications to accommodate more languages will be minimal.

## Availability and requirements

The client, worker and master mGrid toolboxes are made freely available with open source at .

The code distribution toolbox was developed and tested for Matlab version 8, release 14, PHP 4 and Apache HTTP (Web) Server version 2.0.40. The client side toolbox distributed is Operating system independent and uses only native functions of Matlab, itself available for Linux, Solaris, Windows and MacOS [31]. The worker and master server toolboxes were developed for Linux OS. Accordingly, the automatic configuration scripts for those two toolboxes are only guaranteed to work for that choice of OS and web server. However, both Apache and PHP are widely available in equally open source and freely distributed implementations for other operating systems. Similarly, porting the automatic configuration scripts for other web-servers should be straight forward.

## Authors' contributions

YVK performed software development (design, coding, testing, debugging) and elaboration of manuscript. JSA developed the original concept and drafted the implementation layout.
